# Imperceptible, designable, and scalable braided electronic cord

**DOI:** 10.1038/s41467-022-34918-x

**Published:** 2022-11-19

**Authors:** Min Chen, Jingyu Ouyang, Aijia Jian, Jia Liu, Pan Li, Yixue Hao, Yuchen Gong, Jiayu Hu, Jing Zhou, Rui Wang, Jiaxi Wang, Long Hu, Yuwei Wang, Ju Ouyang, Jing Zhang, Chong Hou, Lei Wei, Huamin Zhou, Dingyu Zhang, Guangming Tao

**Affiliations:** 1 Wuhan National Laboratory for Optoelectronics and School of Computer Science and Technology, Huazhong University of Science and Technology, 430074 Wuhan, China; 2 School of Mechanical Engineering and Electronic Information, China University of Geosciences (Wuhan), 430074 Wuhan, China; 3 School of Optical and Electronic Information, Huazhong University of Science and Technology, 430074 Wuhan, China; 4 School of Electrical and Electronic Engineering, Nanyang Technological University, 50 Nanyang Avenue, Singapore, 639798 Singapore; 5 State Key Laboratory of Material Processing and Die & Mould Technology, School of Materials Science and Engineering, Huazhong University of Science and Technology, 430074 Wuhan, China; 6 Wuhan Jinyintan Hospital, 430048 Wuhan, Hubei China; 7 Hubei Provincial Health and Health Committee, 430015 Wuhan, Hubei China

**Keywords:** Electrical and electronic engineering, Computer science

## Abstract

Flexible sensors, friendly interfaces, and intelligent recognition are important in the research of novel human-computer interaction and the development of smart devices. However, major challenges are still encountered in designing user-centered smart devices with natural, convenient, and efficient interfaces. Inspired by the characteristics of textile-based flexible electronic sensors, in this article, we report a braided electronic cord with a low-cost, and automated fabrication to realize imperceptible, designable, and scalable user interfaces. The braided electronic cord is in a miniaturized form, which is suitable for being integrated with various occasions in life. To achieve high-precision interaction, a multi-feature fusion algorithm is designed to recognize gestures of different positions, different contact areas, and different movements performed on a single braided electronic cord. The recognized action results are fed back to varieties of interactive terminals, which show the diversity of cord forms and applications. Our braided electronic cord with the features of user friendliness, excellent durability and rich interaction mode will greatly promote the development of human-machine integration in the future.

## Abstract (web summary)

Inspired by the characteristics of textile-based flexible electronic sensors, the authors report a braided electronic cord with a low-cost, and automated fabrication to realize imperceptible, designable, and scalable user interfaces with the features of user-friendliness, excellent durability and rich interaction mode.

## Introduction

Natural User Interface (NUI) based on novel material and Artificial Intelligence (AI) technology is the key to connecting the physical world with the digital space to enrich human life^1^. From traditional human–computer interaction to the Internet of Things (IoT), it is a hot topic for academic investigation with various applications in healthcare^2–5^, motion monitoring^6–10^, robotics^11–14^, and smart homes^15–17^, etc. Sensor devices are important perception units in the intelligent interaction system. To further realize the human-centric high-experience human–computer interaction pattern, it is required to utilize industrial-friendly, portable, and accurate sensors. However, the existing commercial smart devices are either fully or partially composed of planar and rigid materials, which makes people feel the intense presence. Moreover, these devices generally have a hard casing and are obtrusive accessories, which is incompatible with the idea of seamless human–machine integration. To overcome such limitations, flexible electronics are potential materials as novel sensors to innovate the design of sensors for the perception of environments and body signals.

NUI is essentially an invisible user interface that can provide users with a natural human–computer interaction experience. At present, there are many different strategies to implement NUI, one of which is the use of a “reality user interface” (“RUI”), also known as “reality-based interfaces” methods, which are often implemented using wearable devices. RUI presents objects in the real world as “clickable,” so the wearer can click any daily object to make it act as a hyperlink to integrate cyberspace with the real world. Recent advances in flexible electronics offer unique opportunities to design various types of smart device interfaces, which makes them closer to human and more convenient as interfaces to realize the NUI and RUI.

The concept of multifunctional fibers^18,19^ and flexible electronic sensors^20–26^ with flexible mechanics, comfort, and embedded machine learning algorithms^27^ poses a possible solution to address the aforementioned difficulties. In order to serve human life to the greatest extent, a flexible electronic sensor should be ultimately adapted to form factors that humans are living with, such as textiles. Therefore, with the integration of electronics into the daily usage of textile products, textile-based sensors are attractive prospect for smart devices. Based on various transduction mechanisms, textile-based sensors, including capacitance^28–32^, piezoresistivity^33–35^, piezoelectricity^36–39^, and triboelectricity^40–43^, have been reported. Among these sensors, capacitive strain sensors have been widely investigated due to their simple structure, high sensitivity and excellent stability^25^.

Textile-based sensors have been primarily reported in the form of conformal textiles, such as gloves^44,45^, carpets^46^, and various types of clothes^8,47^. Those kinds of textiles with sensing points distributed on the surface represent complex electronic connections and signal processing on huge area coverage. People live in a complex environment, and those textiles are not suitable for all situations. For example, people are not used to wear gloves or socks for particular interactions in a hot summer. It is challenge to realize high-experienced interactions and practical applications based on efficient pattern and miniaturized sensors. Recently, Google reported an I/O Braid^48^, a tiny interactive textile cord with the embedded spiraling of a repeating braiding topology of touch matrices. The cord shape enables scalable gestures sensing with a small number of sensing channels, and is almost in the form of one dimension. With the development of more complex braided structures (including flat braids, round braids, and braids of three-dimensional structural geometries), a manual crafting technology and an automated processing technology allow for more possibilities in interface design for daily life.

This article presents an imperceptible, designable, and scalable capacitive strain sensor braided electronic cord, formed by core-spun pressure-sensing yarns. The yarns were fabricated by wrapping metal wires with cotton fibers, and spraying polyurethane adhesive to form a stable structure. The resulting cords with thread-like morphology were braided by core-spun pressure-sensing yarns and common yarns into various repeating touch matrices according to different designs. For the design of touch matrices, we fabricated an interactive textile cord with the distribution of spiral channels which can distinguish different positions, different contact areas, and different movements. In addition, based on a variety of braiding technologies, more touch matrices can be included. Without the requirement of huge area coverage, the fine cord was only 2.5 mm in diameter with scalable gestures. This kind of miniaturized interface, which can realize various interaction methods with a small number of channels, can be easily used in various occasions, achieving an imperceptible user experience. Furthermore, some practical applications were also presented to demonstrate the designability and efficiency of this strategy.

## Results

### Braided electronic cord design and fabrication

**Fig. 1:**
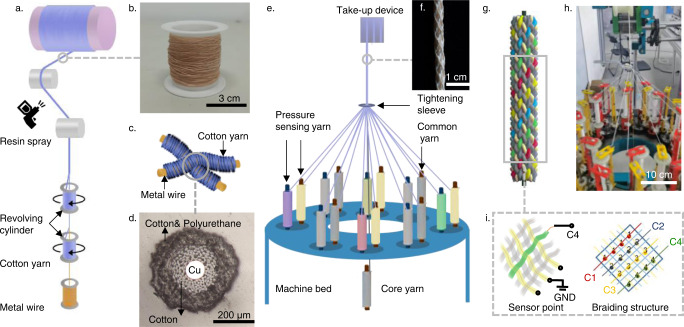
A braided electronic cord based on core-spun pressure-sensing yarns. **a** Schematic illustration for the fabrication of a core-spun pressure-sensing yarn. **b** Photograph of a core-spun pressure-sensing yarn. **c** Schematic illustration for the cross-contact between the core-spun yarns. **d** Optical micrograph of a core-spun yarn cross-section. **e** Schematic illustration for the fabrication of a braided electronic cord. **f** Photograph of a braided electronic cord with a diameter of 2.5 mm. **g** Schematic illustration for the braiding structure. **h** Photograph of the automated fabrication of a braided electronic cord. **i** Schematic illustration for a sensing point and repeating braiding structure of a braided electronic cord.

As shown schematically in Fig. 1a and e, the fabrication approach for producing the braided electronic cords could be realized entirely on the traditional textile process equipment. The procedure involves two main steps: (i) fabrication of a core-spun pressure-sensing yarn; and (ii) fabrication of a braided electronic cord. Based on the capacitive sensing mechanism, a core-spun pressure-sensing yarn consists of a dielectric layer and an electrode layer. The core metal wires were double-wrapped tightly by cotton fibers with revolving cylinders. The double processing provides a protective layer for avoiding exposure of the metal wire. However, the resulting with spiraling stress and fibrous morphology in the cotton layer was unstable due to several sources of deformation, such as bending, pressing, and pinching, which would affect the reliability and accuracy. A stable structure could be achieved by spraying the polyurethane adhesive after the double wrapping, and then being cured in a heating chamber. In fact, the polyurethane adhesive^49^ has been used to bond textiles, taking advantage of its polar groups (–NCO) it contains. These polar groups ensure excellent adhesion for some materials and allow high reactivity with groups containing active hydrogen (–OH, –NH_2_, –COOH, etc.). Figure 1b exhibits scalable yarns fabricated by the traditional textile process. A corresponding higher magnification image of a typical core-spun yarn cross-section with a diameter of 440 μm was exhibited in Fig. 1d. The dielectric layer was made of cotton and the polyurethane adhesive, wrapped conducting metal layer well and tightly. The outermost cotton was permeated by the polyurethane adhesive to tightly wrap the inner structure, which is beneficial to protect the stability of the yarn.

The above steps provide a foundation of capacitive sensing fiber for the follow-up construction of the braided electronic cord. After that, the braiding technology is adopted including automated or manual methods, with core-spun yarns stacked to each other, and a capacitive strain sensing point is formed at the cross-contact point between the two core-spun yarns. The pressure changes the distance between the crossed yarns, which leads to an increase in the capacitance. In the automated processing technology, braiding machines consist of a rectangular or round machine bed on which the bobbins move in a specific way to form a repeating braiding structure. In the manual crafting technology, braiding topology has more combination schemes and complexity. The design of touch matrices in braided electronic cords involves two considerations: (i) common yarns are chosen in addition to core-spun pressure-sensing yarns; and (ii) the repeating braiding structure determines the differences in channel data. Here, the common yarn is a necessary part of realizing human–computer interfaces. Compared to the cord with added common yarns under the same number of channels, the cord made entirely of pressure-sensing yarns with full coverage of sensing points shows no significant differences among various channels when touching different positions, because finger touching covers different channels similarly in the dense sensing points. In other words, the function of added common yarns is to change repeating braiding structure by expanding the structure area or changing the uniformity of the distribution of channels, which would increase the difference between channels under the same area. Besides, the more channels created by core-spun pressure-sensing yarns, the more human–computer interaction data can be obtained by the cord, but at the cost of larger diameter of the cord. In this study, circular braiding was mainly used because of its maturity in the automated processing technology. And the commercial cotton yarn is chosen due to the advantages of low cost, easy access, and maturity in textile processing. As shown in Fig. 1h, commercial yarns and core-spun pressure-sensing yarns are drawn from the bobbins on the corresponding spindles and then interwoven with each other at the tightening sleeve. The reciprocating motion of the spindles from one disc to another on the chassis contributes to the interweaving of yarns around the core yarn. With the help of the top take-up device, the braided electronic cord can be continuously and uniformly wound on the roller without length limitations. Figure 1f shows a typical image of the braided electronic cord with a diameter of 2.5 mm with eight core-spun pressure-sensing yarns and eight commercial cotton yarns. The spiral pattern on the surface is caused by the braided structure and the mechanical difference between the core-spun yarns and the common yarns. The cost of braided electronic cord is very low, as listed in Supplementary Table S1.

To illustrate the structure of this braided electronic cord more directly, Fig. 1i shows the sensing point and the repeating braiding structure of a braided electronic cord. Accordingly, each yarn is distributed along the surface of the braided cord in a spiral path so that the signal distribution of the sensing points in each channel is also spiral. And the adjacent channels are separated by common yarns. The repeating braiding structure is a 4 × 4 array, from which columns are selected as channels. Besides, it is essential to note that other braiding methods commonly have a repeating structure.

### Characterization of braided electronic cord

**Fig. 2:**
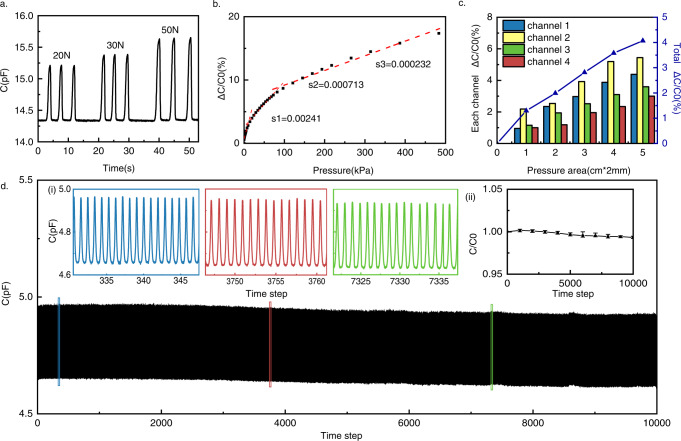
Characterization of braided electronic cord. **a** The capacitance response of the braided electronic cord from a triple compress release cycles of compression under forces of 20 N, 30 N, and 50 N. **b** Relationship between relative change in capacitance and applied pressure of braided electronic cord. **c** Relationship between relative change in capacitance and applied contact area of the braided electronic cord. **d** Capacitance response of braided electronic cord repeated compress release over 10,000 cycles under a force of 35 N (i) and relative capacitance of braided electronic cord without pressure versus the number of repeated compress release cycles (ii).

The metal wires are used as electrodes, with their cotton coating and the spray-coated polyurethane adhesive between them being used as dielectric layers. According to the capacitive sensing principle, their sensing mechanism is as follows: when people press on the cord, the sensing points formed at the cross are deformed by force, and the distance between core-spun yarns reduces, resulting in a significant increase in capacitance. According to this, Fig. 2a shows the capacitance response of the braided electronic cord (length 10 cm) from triple compress release cycles of compression of 20 N, 30 N, and 50 N. Figure 2b shows the capacitive sensing characteristics of a braided electronic cord (length 4.8 cm) while pressed. The sensitivity^28^ of the capacitive pressure sensor can be defined as the slope of the traces in Fig. 2b by three consecutive regions with different values. The sensitivity decreases with increasing pressure, in line with results consistently reported in the literature. For pressures below 14 kPa, between 14 and 97 kPa, and between 97 and 483 kPa, the sensitivities are 0.00241 kPa^−1^, 0.000713 kPa^−1^, and 0.00232 kPa^−1^, respectively. Here, the sensitivity was highest for the lowest applied pressure, which is sufficient for fingers pressing^50^ in human–computer interaction.

In addition to the scale of pressure, the contact area of the pressure was studied by the characteristics of capacitance change. Different sizes of molds were utilized for investigation in this study. The total capacitance of the channels changes linearly with the pressure contact area (Fig. 2c). As the area increases, more sensing points are covered, which is one of the key reasons for judging different gestures (pinching and grabbing). The durability and stability of the braided electronic cord were investigated through repeated loading and unloading cycling tests. As shown in Fig. 2d, the braided electronic cord (length 4.8 cm) exhibits stable output signals in repeated compress release cycles under a force of 35 N. The output signals of the braided electronic cord were stably maintained without any remarkable degradation, despite the intensive cycling tests involving repeating the process over 10,000 cycles. Figure 2d (ii) shows little change in stability from that observed at the initial capacitance value. It can work typically in different ambient temperatures and humidity (Supplementary Fig. S5 and Supplementary Tables S3 and 4), and its washable times >10 times (Supplementary Table S4). Therefore, it can be concluded that the braided electronic cord can be used repeatedly for a long time against the repeated mechanical loads, which is suitable for daily use.

The interactive mechanism of the braided electronic cord is based on structure and characterization. It can recognize the pressing at different positions in a braiding cycle and has similar responses to pressing on the same repeating position in different braiding cycles, as shown in Supplementary Fig. S1. In addition, the braided electronic cord also recognizes the gestures of different contact areas and different pressing actions (Supplementary Fig. S2), which has great application value in human–computer interaction. The interactive mechanism is universal for all cords with repeating braiding structure.

### Cord-based human–computer interaction

**Fig. 3:**
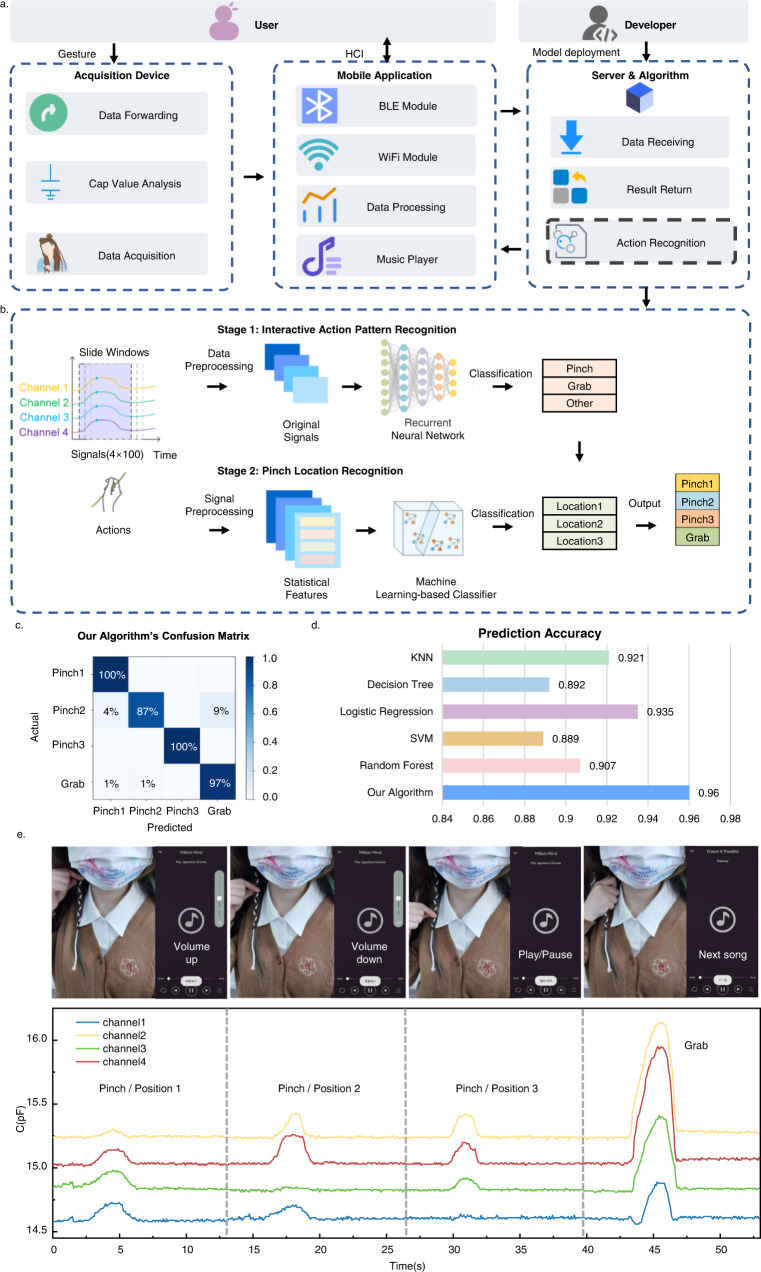
Cord-based human–computer interaction. **a** Processing flow of human–computer interaction of the braided electronic cord. **b** Overview of our proposed algorithm to realize action recognition based on human–computer interaction application. **c** The confusion matrix of our proposed algorithm. **d** Evaluation results of action recognition based on our proposed algorithm and other machine learning-based classifiers. **e** Examples of a miniaturized platform by the braiding cord into hair with three pinches and grab to control music player App, such as switching songs and changing the volume.

To realize the high-experienced human–computer interaction, a cord-based human–computer interaction system is designed as shown in Fig. 3a. Considering the limited computation performance of the mobile phone, we design and develop the software system based on the client or server architecture. The entire system uses modern standard communication methods such as Bluetooth^51^ and Wi-Fi^52^. We used a capacitance acquisition chip with mature technology and connected the circuit. The final acquisition device size is 68 × 68 × 20 mm, and the net weight is 60 g. It can measure capacitance in the range of 0–20pf and has a Bluetooth module, and it can collect data from ten channels simultaneously. First, we connect the braided electronic cord with the micro-capacitor acquisition chip with a Bluetooth communication function to continuously obtain the capacitance characteristics of the braided electronic cord. The chip is connected to the mobile phone through Bluetooth so that the music player App can receive the capacitance value data of the braided electronic cord. We made a simple filter that uses appropriate thresholds to determine the occurrence of actions. Through the statistics of the data set, we set an appropriate threshold. An action is determined only when the value of any channel in the collected data exceeds the threshold. After that, the App gets a generic action recognition model from the server for simple interactive instruction recognition. Due to the different interaction habits of users, it is necessary to collect user interaction data through the App and upload it to the server to update the model. Finally, the App instructs the music player to switch songs and change the volume based on the recognized interaction commands (Supplementary Movie S1).

Based on the interactive mechanism of the braided electronic cord, a specific gesture is applied to the electronic cord, resulting in a unique capacitance data sequence. Since the yarn is woven in a regular way, the features of the data generated by pressing on its different positions have a periodically varying nature. At the same time, when different gestures are used to operate the same position, the data fluctuations are also different. There is a great correlation between the response signals of each channel. Therefore, we collect capacitance values over a period of time as the original time series, and use algorithms to classify actions.

Here, we use a two-stage algorithm to judge the action^53^, as shown in Fig. 3b. In the first stage, the characteristics of the signal and timing information are used to input the recurrent neural network to realize the judgment of the interaction mode. In the second stage, when a “pinch” action is determined, the position is determined according to the characteristic difference of the signal. Finally, we achieved an accurate judgment of the interaction location.

In the first stage, the deep neural network we adopted is a Long Short-Term Memory network and used the interactive action data collected under various temperature and humidity conditions as the training set. Due to the physical structure of the yarn, various statistical features of capacitance fluctuation are different, such as energy, entropy, mean, variance, etc. Therefore, in the second stage, using these statistical features and the filtered capacitance data of each channel, through machine learning methods, the probability that it belongs to each interaction instruction can be obtained. The network can extract and fuse a variety of data features, sequentially capture time series data in the form of sliding windows, and finally give the predicted probability of the action, to obtain the category of the action and the position information of the pressing as shown in Fig. 3d. This algorithm not only pays attention to the characteristics of the signal itself and avoids the loss of features, but also takes advantage of the features of the yarn signal compared with other sensing signals, and designs a feature extraction method that is easier to mine and determine. The two-stage algorithm makes up for the shortcomings of a single algorithm, complements each other in decision-making, and enhances the robustness of the system. At the same time, we also compared with traditional machine learning methods, as shown in Fig. 3c. Our algorithm realizes the recognition accuracy with 0.96. And logistic regression also showed great recognition performance compared with other machine learning-based algorithms. To show the proposed algorithm’s accuracy., the confusion matrix^54^ is obtained, a specific matrix that visualizes the performance of an algorithm. The horizontal axis of the confusion matrix represents the real action, and the vertical axis represents the prediction result. Therefore, the dark color grid on the diagonal represents the prediction accuracy, while other grids represent the prediction error. It can be seen intuitively that the recognition error of the action Pintch2 is the largest, while the recognition accuracy of action Pinch3 is as high as 100%. In Fig. 3d, it is proved that our algorithm can recognize four actions, including three pinches and grab, as it significantly improves the prediction accuracy and reduces misclassification.

**Fig. 4:**
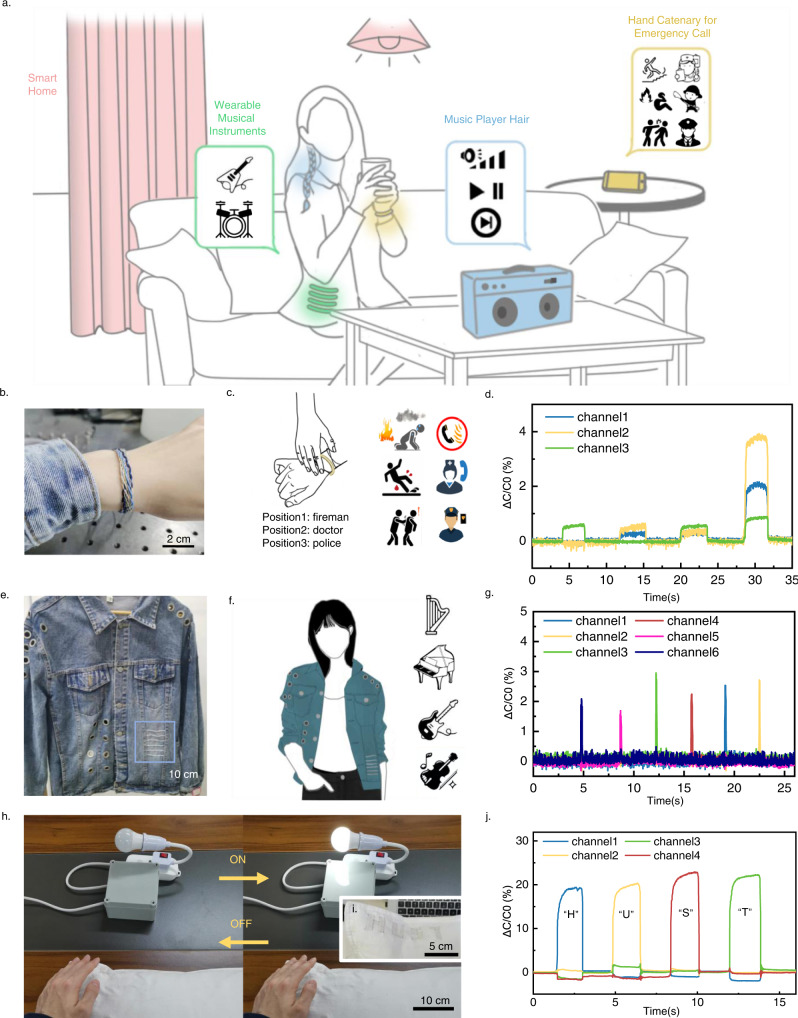
Intelligent interaction application scenario. **a** Schematic illustration for the interaction system based on the braided electronic cord. **b** Photograph of a smart hand catenary. **c** Examples of a smart hand catenary for an emergency call with limited mobility. **d** Capacitance response for different interactive activities to the smart hand catenary. **e** Photograph of smart fabrics based on a knotted cord. **f** Examples of smart fabrics for musical instruments playing. **g** Capacitance response for different interactive activities to the strings. **h** The light can be controlled by pressing interactive embroidery pillow based on core-spun yarns. **i** Photograph of interactive embroidery pillow based on core-spun yarns. **j** Capacitance response of pressing at different letter embroidery.

As the braided electronic cord is a kind of one-dimensional interface with a large aspect ratio and high flexibility, its interaction system has three construction strategies: (i) integrating with flexible or rigid objects in life, (ii) integrating with textile products, and (iii) interacting as an independent flexible device. Taking advantage of the cord form, these strategies cover most situations in daily life, as shown in Fig. 4a.

It was demonstrated that the braided electronic cord could be applied for human–computer interfaces as a miniaturized platform by braiding the cord into hair and testing it with four channels. The capacitance measurement of the hair braid system was carried out by a multichannel capacitance-analyzing circuit system equipped with a Bluetooth module. A dataset for a wearer performing different gestures was captured. The capacitance response of the hair braid in four gestures (pinch position 1, pinch position 2, pinch position 3, and grab) are described in Fig. 3e. When pinching at different positions of the hair braid or grabbing, each channel exhibited a sufficient distinguishable change in capacitance. Because the yarn is cross-braided according to the spiral shape, the contact point and deformation of the yarn are different at various positions, resulting in different capacitance reference values and changes so that it can be distinguished. At the same time, we consider that the recognition accuracy may be reduced after the yarn is rotated. We collected capacitance data from pressing yarn from different angles to enhance the generalization ability of the final algorithm model. We have developed a music player App to show the interaction ability. And the music player App could be successfully controlled with the hair braid system (Supplementary Movie S1).

To prove the feasibility of design in this study, we demonstrated that the electronic cord in other braiding technologies could be applied to many scenes in life. A flat braided electronic cord used as a smart hand catenary to call in case of emergency is shown in Fig. 4c. The hand catenary was made of three core-spun pressure-sensing yarns and three commercial cotton yarns to form a flat intersecting structure. We described the principle of yarn weaving earlier in this paper. It can be known that the more channels, the easier it is to distinguish the capacitance fluctuations of the woven yarn at different positions. Compared with the application scenario shown in Fig. 3, since few operations can be performed on the intelligent hand catenary, we only use three channels designed before the project began. When pressed on different positions or pressed with a palm, the hand catenary had different responses (Fig. 4d), demonstrating the convenience of the cord-based human–computer interface compared to a rigid interface. In some emergency situations, people’s movements may be restricted, such as the need to report to the police when they are beaten, the inability to move when they are injured, or the difficulty in moving when the fire is severe. In such cases, the absence of mobile phones or other rescue devices will lead to severe consequences. Therefore, integrating the emergency call function into the hand catenary is of important value. The three positions on the hand catenary correspond to the three functions of alarm, medical first aid and fire rescue, respectively. The palm press is used to wake up call status to prevent accidental contact in daily life. Due to the softness of textile-based sensors, the existence of a human–computer interface could hardly be felt by users.

Besides the above functions, considering that calls may not be efficient in emergency scenarios, we have made some changes to the app to achieve positioning and SMS sending. We have integrated the location SDK into the app. After detecting the pressing for more than 3 s, the app starts to get the detailed location of the mobile phone, which can be accurate to the specific street. Then press different positions to send the location information to the public security organs through the SMS platform to realize a more convenient and quiet alarm and call for help.

A knotted cord based on two core-spun pressure-sensing yarns can be used in human–computer interfaces. This knotted cord was well integrated into clothes through seaming due to its miniaturized size (Fig. 4e). Single knotted cord can be used for interactive functions as wearable strings (Supplementary Movie S2). Each knotted cord exhibited a sufficiently distinguishable change in capacitance without considerable interference among various cords (Fig. 4g). A guitar App was developed to show its appropriate response. In this case, people can play music freely in daily life anytime and anywhere without a heavy guitar (Fig. 4f). Besides, the core-spun pressure-sensing yarns can also be used as capacitive pressure sensors in a plain structure (Supplementary Fig. S3) or other cross structure. Inspired by the intelligent imperceptible digital ward^55^, we also developed two layers of embroidery on a hospital pillow to help the patients who are limited in movement with the actions of remote control. A patient with limited mobility can touch the embroidery to turn the lamp on and off (Fig. 4h–k and Supplementary Movie S3). It also paves the way for the application prospect of the electronic cord in interactive digital wards.

## Discussion

In summary, we developed an imperceptible, designable and scalable braided electronic cord-based capacitive pressure sensor strategy, formed by core-spun pressure-sensing yarns. Our approach used low-cost and easily available textile and electronic materials in automated fabrication which can be expected to be industrially mass produced and practically applied to people’s lives. The proposed braided electronic cord can recognize gestures with different positions, different contact areas and different movements in a small diameter (2.5 mm), and can withstand at least 10,000 cycles of compress release. Benefitting from the fine diameter, the cord is soft and almost in a one-dimensional form, and cord-based interactive systems have a variety of design strategies. Cord-based human–computer interactions show the diversity of braided electronic cord design and application, which is suitable for most situations in daily life, and will promote the developments of human–machine integration. In the future, the cord-based interfaces can be widely deployed everywhere to construct NUI-based physical world and digital world. It will greatly make human enjoy the intelligent interaction brought by novel material and AI technology.

## Methods

### Materials

The materials of core-spun pressure-sensing yarn and braided electronic cord are described in the following sections.

#### Core-spun pressure-sensing yarn

The cotton fibers and the polyurethane adhesive were used to form the dielectric layer. The core metal wires were copper in 0.1 mm diameter to form a conductive layer. The polyurethane adhesive TS-8217 was obtained from Dongguan Yantai Chemical Technology Co., LTD to form a stable capacitance structure.

#### Braided electronic cord

The common polyester yarns were chosen in addition to core-spun pressure-sensing yarns to be braided together. The core yarns were nylon to construct stable solid rather than hollow structures.

### Fabrication of core-spun pressure-sensing yarn

The copper wires and cotton fibers are respectively guided into the special yarn tube of the automated braiding machine through the precision winder (Zhejiang Weifeng Machinery Co., Ltd., WF55C). The yarn tube is assembled on the automated covering machine (Zhejiang Weifeng Machinery Co., Ltd., WF288), and copper wire is the core wrapped with two layers of cotton fibers. The polyurethane adhesive is sprayed on the surface of wrapped yarn with pneumatic spray gun, and then it is fully cured in a heating chamber (Shanghai Yiheng Scientific Instrument Co., Ltd., DZF-6090) at 50 °C for 24 h.

### Fabrication of braided electronic cord

The automated braiding machine (Xuzhou Juzheng Machinery Co., Ltd., 90/32) is equipped with eight groups of polyester yarns, eight groups of core-spun yarns, and a group of nylon core yarn. Among the core-spun yarns, the four groups and the remaining four groups are respectively installed on both sides of the impeller to ensure effective interweaving of core-wrapped yarns in the final braiding structure because both sides of the spindle of the same impeller move in opposite directions.

### Characterization testing

The ends of core-spun yarns were immersed in dimethylformamide (DMF) solution, and the surface cotton fibers were untwisted after the surface polyurethane adhesive was dissolved to obtain bare copper wire. A multichannel capacitance-analyzing circuit with a Bluetooth module was designed and manufactured by Linkzill (Hangzhou, China). A mechanical testing machine (Dongguan Zhiqu Precision Instrument Co., Ltd., ZQ-990B) was used to apply precisely controlled force onto the braided electronic cord.

### Design of the music player App

The music player App is developed using Android Studio. The functional modules are described in the following sections.

#### Bluetooth communication module

It is responsible for communicating with the capacitor acquisition chip, realizes full support for the communication interface of the acquisition chip, and transmits the collected data directly to the data module.

#### The data analysis module (core module)

It implements all data processing and analysis functions, including data formatting, dynamic adjustment of capacitance reference values, and fluctuation detection (only when fluctuations above the threshold are detected, we consider that the user has taken action, it can reduce the communication overhead of Wi-Fi), forwarding the data to the server, music player control (converting the recognition result into the corresponding operation of the App).

#### Wi-Fi communication module

It is responsible for communication with the server, mainly for sending raw data.

#### Music player module

It realizes local music file scanning, music playback, player control (switching songs, volume control, music playing, and pause).

### Design of the alarm App

The alarm App is implemented in a similar way to the music player. It also has a Bluetooth communication module to obtain the capacitance value data of the bracelet through the capacitance acquisition chip. The user’s operation on the bracelet is relatively simple, so the action recognition algorithm is directly integrated into the App without connecting to the server for recognition. At the same time, the alarm App integrates the Baidu Map SDK, which can obtain the specific geographic location of the user. When the user triggers the alarm, the App will send the location information to the alarm platform via SMS.

## Supplementary information

Supplementary Information

Description of Additional Supplementary Files

Supplementary MovieS1

Supplementary MovieS2

Supplementary MovieS3

## Data Availability

The data in plots generated in this study are provided in the Supplementary Source Data file and the dataset used in this study are available in the GitHub database [https://github.com/fabricComputing/braidedElectronicCord]. Source data are provided with this paper.

## Source data

Source Data

## Code availability

Custom code and dataset used in the current study are available in the GitHub database [https://github.com/fabricComputing/braidedElectronicCord].
