# Supplementary material for: Imperceptible, designable, and scalable braided electronic cord
Source: Nat Commun. 2022 Nov 19;13:7097. doi: 10.1038/s41467-022-34918-x (PMC9675780; doi:10.1038/s41467-022-34918-x)
Supplement: Supplementary file 1 — Supplementary Information [file 41467_2022_34918_MOESM1_ESM.pdf]

Supplementary Information for

Imperceptible, Designable, and Scalable Braided Electronic Cord

Min Chen[1]†, Jingyu Ouyang[1]†, Aijia Jian[1]†, Jia Liu[1]†, Pan Li[1]†, Yixue Hao[1]†, Yuchen Gong[1], Jiayu Hu[1], Jing Zhou[1], Rui Wang[1], Jiaxi Wang[1], Long Hu[1], Yuwei Wang[1], Ju Ouyang[1], Jing Zhang[2], Chong Hou[1, 3], Lei Wei[4], Huamin Zhou[5], Dingyu Zhang[6,7], Guangming Tao[1, 5]*

1. Wuhan National Laboratory for Optoelectronics and School of Computer Science and Technology, Huazhong University of Science and Technology, Wuhan 430074, China

2. School of Mechanical Engineering and Electronic Information, China University of Geosciences (Wuhan), Wuhan 430074, China

3. School of Optical and Electronic Information, Huazhong University of Science and Technology, Wuhan 430074, China

4. School of Electrical and Electronic Engineering, Nanyang Technological University, 50 Nanyang Avenue, Singapore 639798, Singapore

5. State Key Laboratory of Material Processing and Die & Mould Technology, School of Materials Science and Engineering, Huazhong University of Science and Technology, Wuhan 430074, China

6. Wuhan Jinyintan Hospital, Wuhan 430048, Hubei, China

7. Hubei Provincial Health and Health Committee, Wuhan 430015, Hubei, China

†These authors contributed equally to this work.

*Corresponding author. Email: tao@hust.edu.cn

Table of Contents

1. Supplementary Discussions

1.1 Fabrications for knotted cords and hand catenary (Page 1)

1.2 The interactive mechanism of recognizing different positions (Page 1)

1.3 The interactive mechanism of recognizing different contact areas (Page 1)

1.4 The interactive mechanism of recognizing different movements (Page 2)

1.5 The plain textile based on core-spun yarns (Page 2)

1.6 Cost estimation (Page 2)

1.7 The interaction performance under temperature and humidity environment (Page 3)

1.8 Washability of the braided electronic cord (Page 3-4)

2. Supplementary Table

Table S1 (Page 5)

Table S2 (Page 5)

Table S3 (Page 5)

Table S4 (Page 6)

3. Supplementary Figures

Figures S1-4 (Page 7-8)

1. Supplementary Discussions

1.1 Fabrications for knotted cords and hand catenary

With the development of braiding technology, There are many braiding strategies such as twist, knot, round braid, square braid, flat braid, and 3D braid. These braided cords have the repeating braiding structure in common, so the interactive mechanism discussed in this article is universal for them. Therefore, the forms of cord-based human-computer interaction are varied.

The knotted cords used in smart fabrics for musical instruments playing consist of two core-spun pressure sensing yarns, and the knots are made by intertwining the yarns, resulting the capacitive strain sensing points. The smart hand catenary for an emergency call with limited mobility consists of 6 core-spun pressure sensing yarns and 6 commercial cotton yarns through flat braiding.

1.2 The interactive mechanism of recognizing different positions

The finger touches a part of the braided electronic cord, covering the sensing points of each channel. Since the sensing points of each channel on the braided electronic cord are not uniformly distributed, there will be differences in the number of sensing points covered by fingers and the force between each channel. Based on the repeating spiral distribution of channels, pressing on the different positions in the same braiding cycle obtains the differences in channels response, representing the features of positions (Figure S1a). Pressing on the same repeating position in different braiding cycles obtains the similar channels response (Figure S1b).

1.3 The interactive mechanism of recognizing different contact areas

When the pressure contact area changes, the number of sensing points covered by the area will change linearly (Figure S2b). Different gestures have different contact areas, so this characteristic can be applied to gesture determination of human-computer interaction. At the same time, we also need to note that the change of the total capacitance value is not only related to the pressure contact area, but also the scale of

pressure. Therefore, gestures with large differences in the pressing contact area should be selected for interaction in order to ensure the accuracy of the interaction. In other words, a small-area gesture must use a great force to achieve the capacitive response range that a large-area gesture can achieve, or even cannot reach the capacitive response of a large-area gesture. Based on this rule, misidentifications can be avoided in daily usage habits.

1.4 The interactive mechanism of recognizing different movements

The braided electronic cord can also recognize different movements, through the waveform characteristics of the capacitive response changing with time in dynamic gestures. To illustrate this interactive mechanism, three typical movements are used to interact with the braided electronic cord, and the capacitance response is shown in Figure S2c. The waveforms from left to right are slide, long press, and tap at braided electronic cord. The capacitive response of slide appears as a fluctuating curve with multiple extremas, and the location of the extremas varies from channel to channel. The capacitive response of long press appears as a square wave-like waveform. The capacitive response of tap appears as two continuous sharp peaks.

1.5 The plain textile based on core-spun yarns

In addition to braiding technology, the core-spun yarns can also be used to weave plain fabrics as capacitive strain sensors. Figure S3a and Figure S3b show the measurement of static and dynamic forces, Figure S3c shows the good durability of the plain textile.

1.6 Cost estimation

According to the optical micrograph of a core-spun yarn cross-section, we assume the diameter of the polyurethane adhesive layer and the inner structure are 440 and 270 μm (Figure 1d), respectively. Therefore, we consume about 9.48 cm^3 polyurethane adhesive for every 100 m of core-spun yarn. Cost estimation for braided electronic cord is shown in Supplementary Tables 1.

1.7 The interaction performance under temperature and humidity environment

The test of temperature effect on interactive pattern recognition. We tested the electronic braided cord (length 133 cm) in a constant temperature and humidity chamber (MC-800C). Keeping the humidity stable at 60%, its internal temperature gradually increased from -10 °C to 40 °C, each time increased by 5 °C. with the change of temperature from -10 ℃ to 40 ℃, the capacitance change range of the electronic cord is within 2.581%. We collected 160 sets of data at each temperature, under different actions, such as "long press", "swipe", "pinch position 1", "pinch position 2", "pinch position 3", "twist", "double click" ", and "grab". The algorithm extracts signal features such as energy, entropy, mean, variance, etc., then uses a machine learning model for action classification. Recognition accuracy for 8 interactive actions "long press", "swipe", "pinch position 1", "pinch position 2", "pinch position 3", "twist", "double click", and "grab", as shown in the Table S2.

The test of humidity effect on interactive pattern recognition. The electronic braided cord (length 133 cm) was tested in a constant temperature and humidity chamber (MC-800C), keeping the temperature stable at 25°C, and increasing its internal humidity from 15% to 85%, 5% each time. In figure S5b, the results indicate that the overall fluctuation error of the capacitance value is within 0.52% with the increase in humidity. We collected 160 sets of data at each humidity, under different actions, such as "long press", "swipe", "pinch position 1", "pinch position 2", "pinch position 3", "twist", "double click" ", and "grab". The algorithm extracts signal features such as energy, entropy, mean, variance, etc., then uses a machine learning model for action classification. Recognition accuracy for 8 interactive actions "long press", "swipe", "pinch position 1", "pinch position 2", "pinch position 3", "twist", "double click", and "grab", as shown in the Table S3.

1.8 Washability of the braided electronic cord

Data were collected in the water washing experiment. An interactive action classification model was trained each time the electronic braided cord was put into the washing machine after 42 minutes of machine washing and air drying. The same

classification model was used to classify 5 interactive patterns ("press or slide", "pinch", "twist", "double-click", and "grab") to identify, and the interaction accuracy is calculated. As can be seen from washability of braided electronic cord is shown in Table S4., after 10 times of water washing, the interactive performance has no obvious effect.

2. Supplementary Table

Table S1: Cost estimation for braided electronic cord in this work.

Materials	Unit price	Total price
Cotton fiber	$0.009/100m	$0.113/100m
Copper wire	$9.126/100m	NA
Polyurethane adhesive	$15.444/1L	$0.146/100m
Core-spun yarn	$9.385/100m	$150.16/100m
Polyester	$0.023/100m	$0.372/100m
Nylon	$0.936/100m	NA
Braided electronic cord	$151.468/100m	NA
A smart hair braid	NA	$0.454

Table S2: The 8 interactive action recognition accuracy of electronic braided cord using the adaptive model at different temperature.

**Temperature**	-10℃	0℃	10℃	20℃	30℃	40℃	50℃
**Accuracy**	94.64%	96.08%	98.75%	98.56%	98.11%	97.92%	98.11%

Table S3: The 8 interactive action recognition accuracy of electronic braided cord using the adaptive model at different humidity.

**Humidity**	20%	40%	60%	80%
**Accuracy**	96.15%	98.04%	98.98%	98.04%

Table S4: Washability of the braided electronic cord in this work.

**Washability**	1st	2nd	3rd	4th	5th
**Accuracy**	0.95	0.9375	0.95	0.9625	0.9375
**Washability**	6th	7th	8th	9th	10th
**Accuracy**	0.9875	0.9583	0.9625	0.9375	0.9375

3. Supplementary Figures

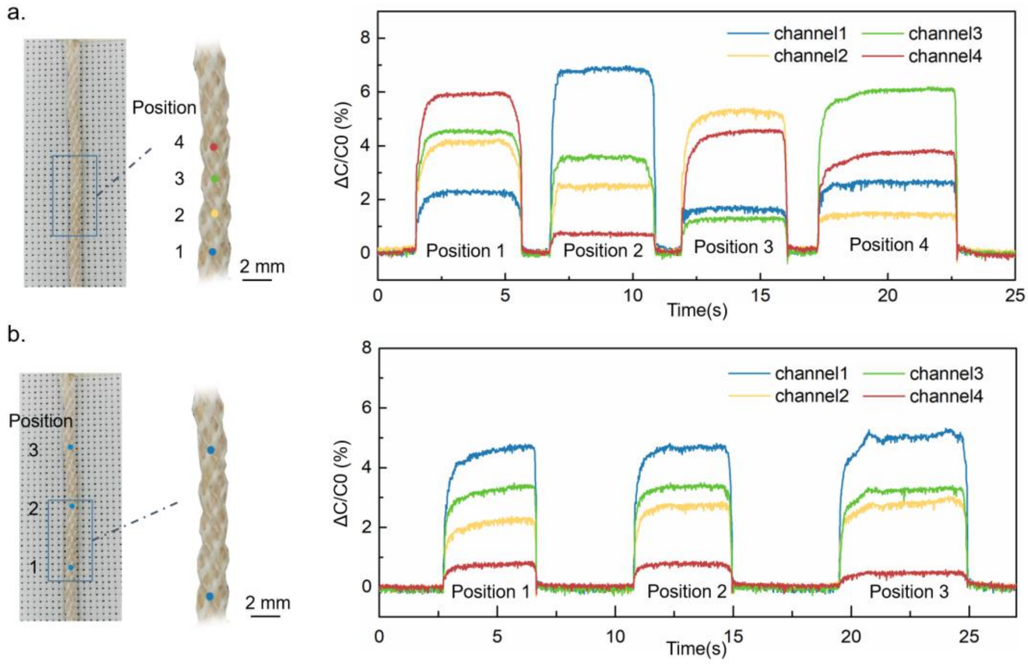

Figure S1. Capacitance response of braided electronic cord when pressed at different positions. a) Different positions in the same braiding cycle. b) Similar repeating positions in different braiding cycles.

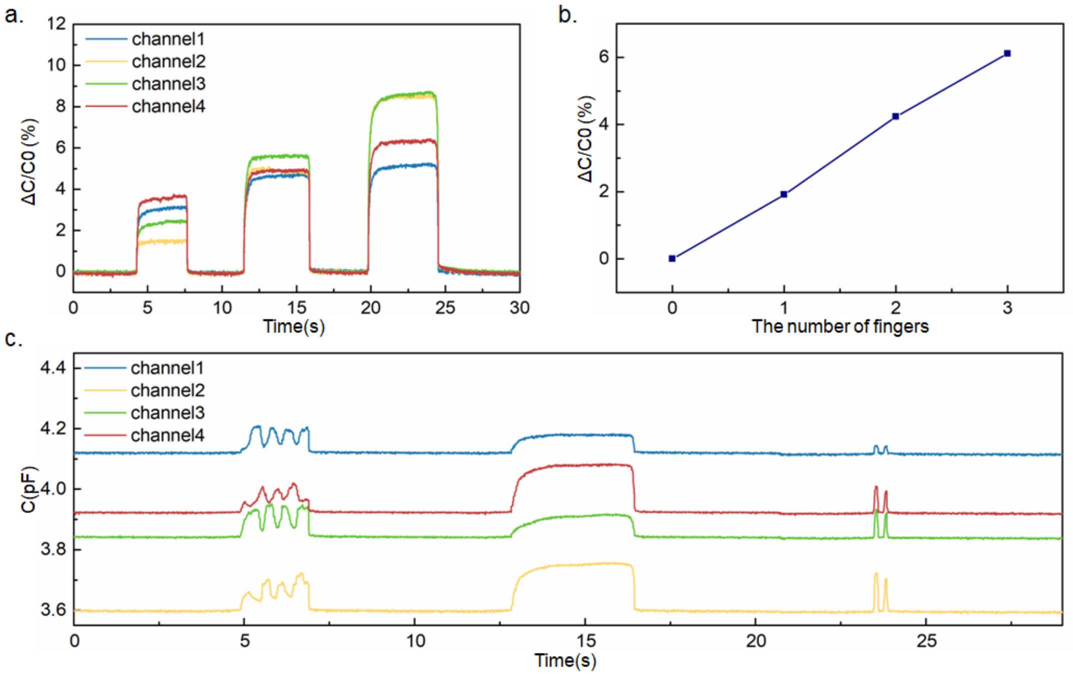

Figure S2. Capacitance response of braided electronic cord when pressed at different contact areas and movements. a) From left to right is the capacitance response of each channel when 1-3 fingers are pressing. b) The total capacitance response of the channels with different number of fingers. c) Slide, long press, and tap

at braided electronic cord.

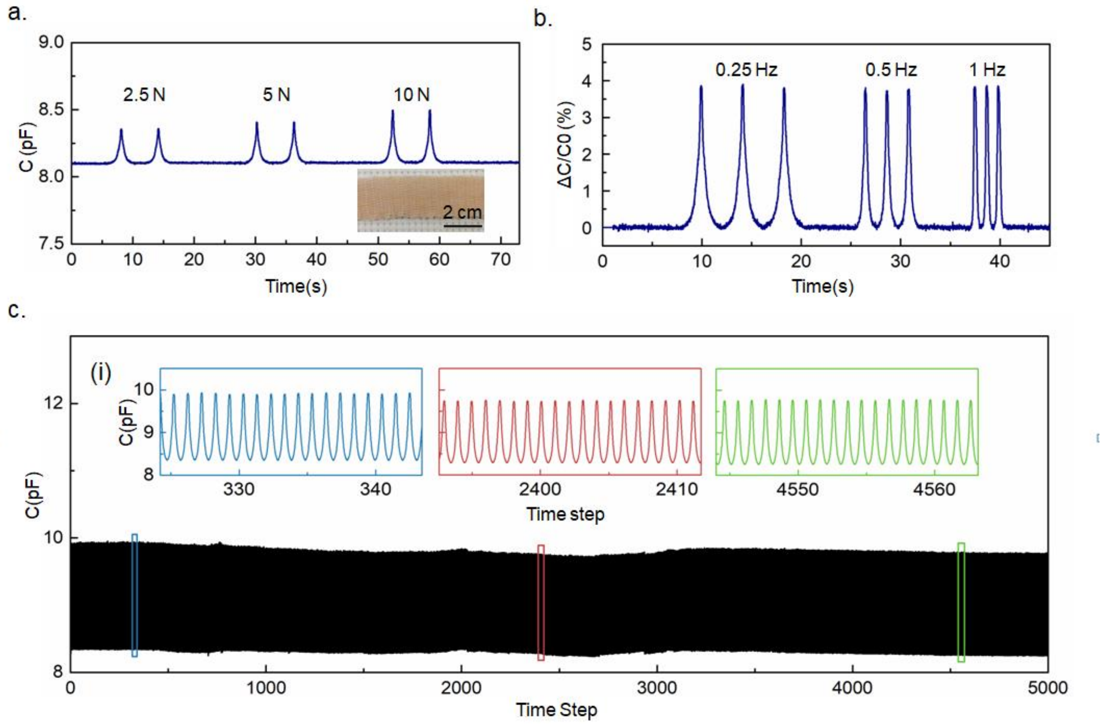

Figure S3. Plain textile based on core-spun yarns. a) The capacitance response of pressure sensing textile pressed at 2.5 N, 5 N, and 10 N, respectively. b) The capacitance response of pressure sensing textile pressed at 0.25 Hz, 0.5 Hz, 1 Hz, respectively. c) Capacitance response of pressure sensing textile to repeated compress release cycles under 35 N and the details (i).

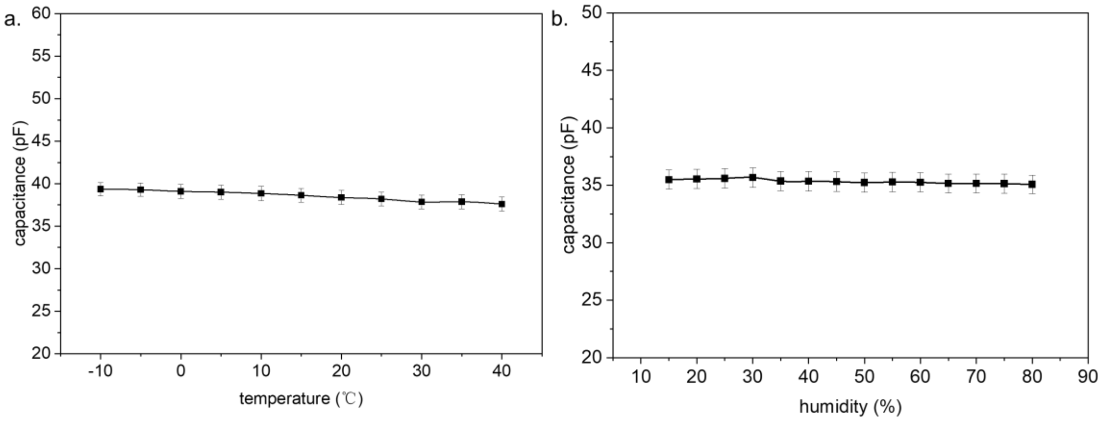

Figure S4. The temperature and humidity effect of electronic braided cord a) The relationship curve of capacitance of electronic braided cord with temperature. b) The relationship curve of capacitance of electronic braided cord with humidity.
