# Supplementary material for: Imperceptible, designable, and scalable braided electronic cord
Source: Nat Commun. 2022 Nov 19;13:7097. doi: 10.1038/s41467-022-34918-x (PMC9675780; doi:10.1038/s41467-022-34918-x)
Supplement: Supplementary file 2 — Description of Additional Supplementary Files [file 41467_2022_34918_MOESM2_ESM.docx]

**Description of Additional Supplementary Files**

**File Name: Supplementary Movie 1
Description:** A miniaturized platform by the braiding cord into hair.

**File Name: Supplementary Movie 2
Description:** Wearable strings based on single knotted cord.

**File Name: Supplementary Movie 3
Description:** Interactive embroidery pillow based on core-spun yarn.
