# Supplementary figures and images for: Imperceptible, designable, and scalable braided electronic cord
Source: Nat Commun. 2022 Nov 19;13:7097. doi: 10.1038/s41467-022-34918-x (PMC9675780; doi:10.1038/s41467-022-34918-x)

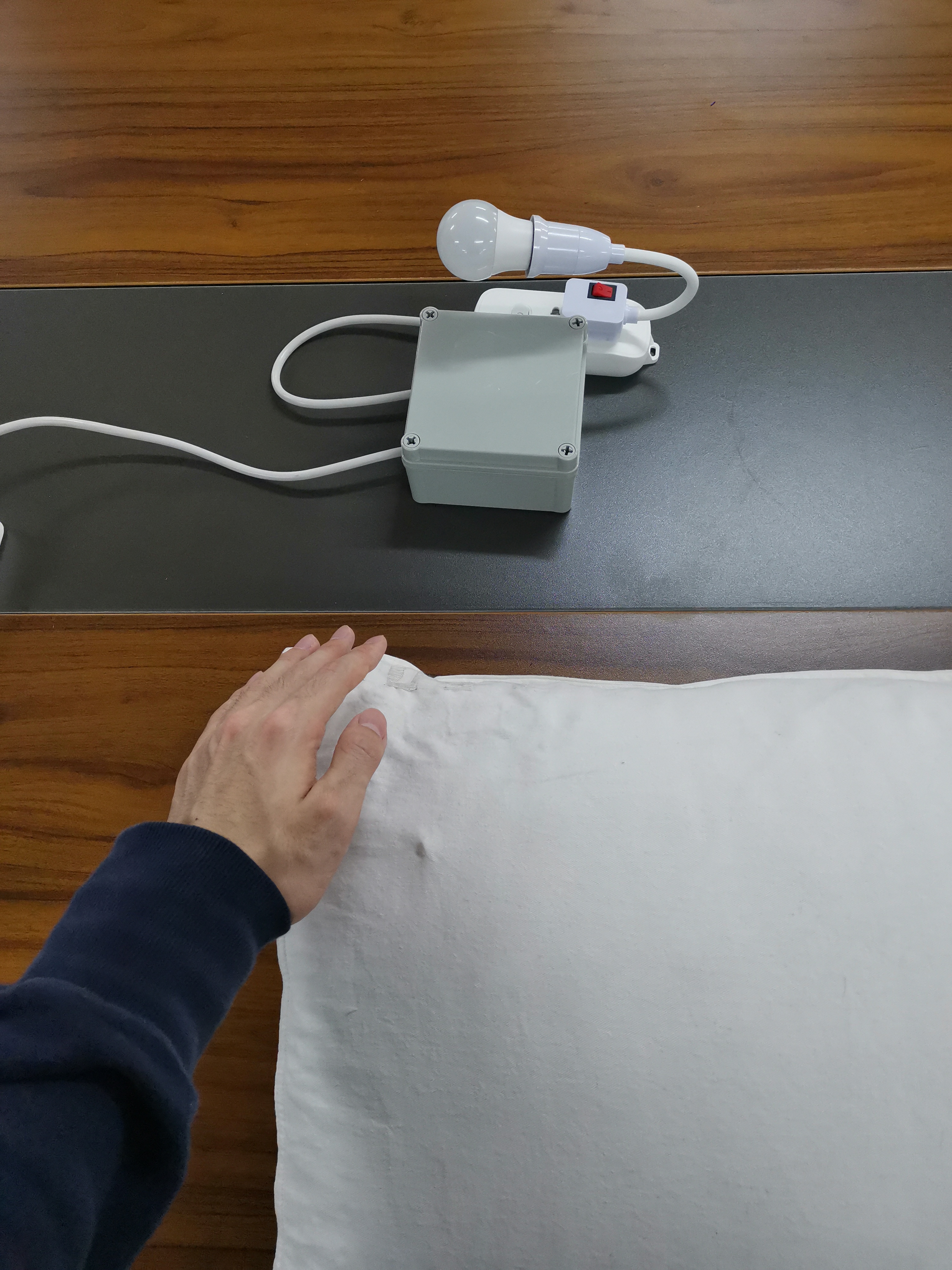

Supplement: Supplementary file 6 — Source Data [file 41467_2022_34918_MOESM6_ESM.zip › Supplementary Source Data/figure4j pillow/lamp off.jpg]

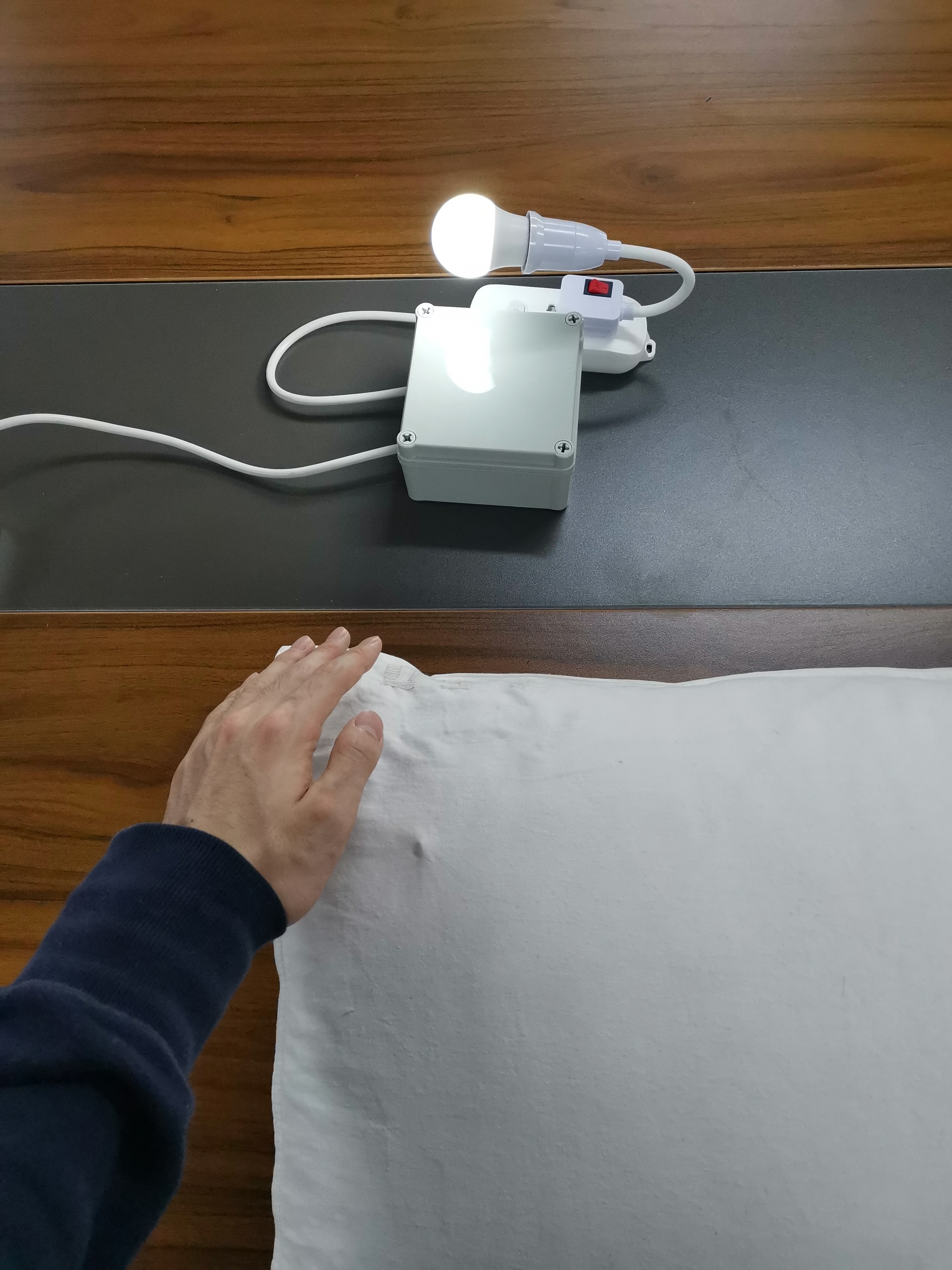

Supplement: Supplementary file 6 — Source Data [file 41467_2022_34918_MOESM6_ESM.zip › Supplementary Source Data/figure4j pillow/lamp on.jpg]
